# Impact of Shunt-Related Hemorrhage on Seizure Development After Ventricular Shunt Surgery in Idiopathic Normal Pressure Hydrocephalus

**DOI:** 10.1227/neuprac.0000000000000171

**Published:** 2025-10-02

**Authors:** Munetake Yoshitomi, Takahisa Nonaka, Ryusei Nobori, Keiichiro Furuta, Naohisa Miyagi, Yuji Okamoto, Kazunori Kajihara, Kenta Murotani, Motohiro Morioka

**Affiliations:** * Department of Neurosurgery, Yayoigaoka Kage Hospital, Tosu, Japan;; ‡Department of Neurosurgery, Saiseikai Yahata General Hospital, Kitakyusyu, Fukuoka, Japan;; § Department of Neurosurgery, Kurume University School of Medicine, Kurume, Japan;; ‖ Biostatistics Center, Kurume University, Kurume, Japan

**Keywords:** Ventricular shunt, Shunt-related hemorrhage, Seizures

## Abstract

**BACKGROUND AND OBJECTIVE:**

Ventricular shunt surgery, with either ventriculoperitoneal (VP) or ventriculoatrial (VA) shunts, is a primary treatment of idiopathic normal pressure hydrocephalus. However, postoperative seizures can complicate recovery. In this study, we investigated risk factors of postoperative seizures and assessed the risk of shunt-related hemorrhage.

**METHODS:**

Patients who underwent VP or VA shunt surgery for idiopathic normal pressure hydrocephalus between April 2020 and March 2023 were retrospectively reviewed. Exclusion criteria included a history of epilepsy or shunt revision surgery. Collected data included patient demographics, incidence of shunt-related intracranial hemorrhage, antithrombotic use, preoperative international normalized ratio, platelet count (within 2 weeks preoperatively), postoperative CRP levels (on day 1), time to seizure onset, and previous intracranial hemorrhage, cerebral infarction, or neurosurgery. Follow-up duration was recorded.

**RESULTS:**

This study enrolled 185 patients (102 men) with a mean age of 79.2 years (range: 53-94). Of these, 153 and 32 patients underwent VP and VA shunt surgery, respectively. Intracranial hemorrhage history was present in 9 patients (4.8%), cerebral infarction in 43 (23.2%), and previous intracranial surgery in 9 (4.8%). Hypertension was noted in 91 patients (49.1%). The mean CRP level on postoperative day 1 was 2.01 mg/dL (range: 0.05–14.99), whereas the mean preoperative international normalized ratio was 1.03 (range: 0.11-1.68). Shunt-related intracranial hemorrhage occurred in 14 patients (7.5%) and seizures in 8 (4%). Antithrombotic agents were used in 32 patients (17.2%). Patients with seizures had a higher incidence of shunt-related hemorrhage. Older age, previous intracranial surgery, and antithrombotic use were significantly associated with shunt-related hemorrhage. Logistic analysis identified shunt-related hemorrhage as a risk factor of seizures up to 2 years postoperatively.

**CONCLUSION:**

Our findings underscore the significance of shunt-related intracranial hemorrhage as a contributing factor to seizures after ventricular shunt surgery. This risk should be clearly communicated to patients during the informed consent process.

## Graphical abstract

**Figure:**
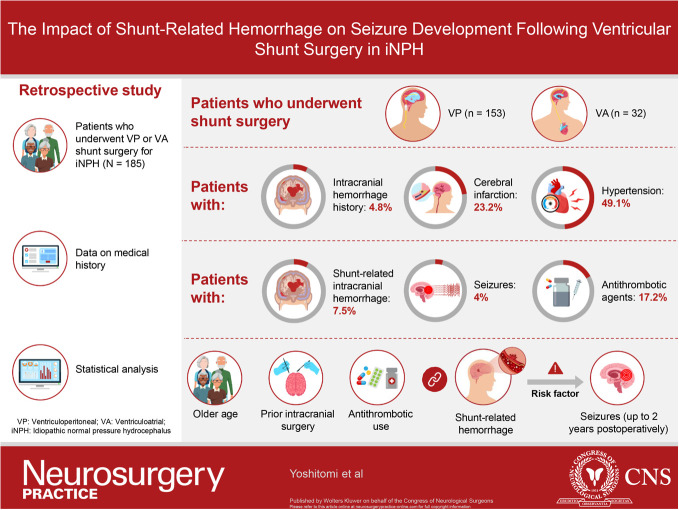

**ABBREVIATIONS:**
APTT: activated partial thromboplastin time
CI: cerebral infarction
iNPH: idiopathic normal pressure hydrocephalus
INR: international normalized ratio
POD1: postoperative day 1
SAH: subarachnoid hemorrhage
VA: ventriculoatrial
VP: ventriculoperitoneal
VSRH: ventricular shunt–related hemorrhage.

Idiopathic normal pressure hydrocephalus (iNPH) is a common condition characterized by the accumulation of cerebrospinal fluid in the brain, leading to symptoms such as gait disturbance, urinary incontinence, and cognitive decline, particularly in older adults. Ventriculoperitoneal (VP) and ventriculoatrial (VA) shunt surgeries are common treatment options for iNPH, as they facilitate cerebrospinal fluid drainage. However, these procedures carry a risk of complications, with seizures being a notable concern.^1^

Although seizures are recognized as an important postsurgical complication, the risk factors contributing to their onset remain unclear. In addition, postoperative intracranial hemorrhage after shunt surgery has been identified as a potential complication that may influence seizure occurrence.

Despite reports of seizures and intracranial hemorrhage after shunt surgery, the underlying mechanisms and risk factors remain insufficiently explored.^1-4^ Specifically, the relationship between shunt-related hemorrhage and seizure development warrants further investigation. Furthermore, the impact of antithrombotic therapy, along with patient's history of stroke or intracranial surgery, on increasing the risk of these complications is not fully understood. Therefore, the aim of this study was to evaluate the association between postoperative intracranial hemorrhage and seizures in patients with iNPH undergoing shunt surgery to identify key risk factors.

## METHODS

This retrospective case-control study was approved by the ethics committee of the institution. The requirement for written informed consent was waived because of the retrospective study design.

### Patients

This study retrospectively analyzed patients with iNPH who underwent ventricular shunt surgery (either VP or VA shunt) at our institution between April 2020 and March 2023. Patients with a history of epilepsy or those who underwent shunt revision surgery or lumboperitoneal shunt were excluded.

### Ventricular Shunt Surgery

All patients were diagnosed with iNPH according to established guidelines^5,6^ and underwent shunt surgery based on their response to the cerebrospinal fluid tap test. The choice of operative procedure, either VA or VP, was determined by patient-specific conditions, such as a history of previous abdominal surgery. All shunt tube insertions were performed by a frontal approach. Programmable valve systems were used in all cases.

### Data Collection

The following patient data were retrospectively collected from the medical records: sex, age, shunt-related complications (including the occurrence of intracranial hemorrhage associated with shunt placement), use of antithrombotic drugs (antiplatelet or anticoagulant), international normalized ratio (INR), platelet count within 2 weeks before surgery, CRP level on postoperative day 1 (POD1), seizure occurrence (including the time from surgery to seizure onset), and medical history of intracranial hemorrhage (>24 hours later from ventricular shunt surgery), cerebral infarction, or previous intracranial surgery.

### Statistical Analysis

Univariate analysis was performed using the χ^2^ or Wilcoxon rank sum tests to compare binary or non-normally distributed continuous variables between patients with and without seizures, as well as between patients with and without intracranial hemorrhage associated with shunt placement. Statistical significance was set at *P* < .05. All statistical analyses were conducted using JMP Pro 16.0 (SAS Institute).

## RESULTS

This study included 185 patients, including 102 men and 83 women, with a mean age of 79.2 years (median: 80 years; range: 53-94 years). Among these patients, 153 underwent VP shunt surgery, whereas 32 underwent VA shunt surgery. Assessment of patient medical history revealed that 9 patients (4.8%) had a history of intracranial hemorrhage, 43 patients (23.2%) had a history of cerebral infarction, 9 patients (4.8%) had a history of intracranial surgery, and 91 patients (49.1%) had a history of hypertension. Postoperative CRP levels on POD1 showed a mean value of 2.01 mg/dL (median: 1.4 mg/dL; range: 0.05-14.99 mg/dL). Preoperative INR had a mean value of 1.03 (median: 1.02; range: 0.11-1.68), whereas activated partial thromboplastin time (APTT) had a mean of 32.5 seconds (median: 32.8 seconds; range: 22.1-51.7 seconds). Fourteen patients experienced postoperative intracranial hemorrhage (7.5%), whereas 8 patients (4%) had seizures. No significant subdural hematomas were observed postoperatively in any of the cases. All postoperative intracerebral hemorrhages were associated with the shunt tube tract. The timing of hemorrhage onset varied widely, ranging from 1 to 664 days postoperatively. Most cases (n = 12) occurred during the subacute recovery phase (1-30 days postoperatively), whereas 1 case presented as delayed hemorrhage (>30 days postoperatively). A history of antithrombotic drug use was recorded in 32 patients (17.2%). When comparing patients with and without seizures, those with seizures had a significantly higher incidence of shunt-related intracranial hemorrhage (Table 1). In addition, patients with and without shunt-related intracranial hemorrhage differed significantly in age (older age), history of intracranial surgery, and use of antithrombotic agents (Table 2). The mean duration from ventricular shunt surgery to the onset of epileptic seizures was 223 days (median: 162 days; range: 1-664 days). Shunt-related cerebral hemorrhage occurred at a mean of 56 days postoperatively (median: 7 days; range: 1-664 days). In the most protracted case, shunt-related hemorrhage triggered the onset of epileptic seizures approximately 2 years postoperatively (Figure). In this study, we reviewed cases involving ventricular shunt–related hemorrhage (VSRH), summarizing patient demographics, clinical history, and relevant surgical details. The cohort included 13 patients, aged between 69 and 89 years. All patients had undergone either ventriculoperitoneal or ventriculoatrial shunt placement. The summary includes data on preoperative platelet counts, INR/APTT, antithrombotic use, hemorrhage onset, and type of VSRH, which primarily involved cortical hemorrhage, intraventricular hemorrhage, and subarachnoid hemorrhage. The clinical outcomes also noted the presence or absence of seizures and their onset postsurgery (Table 3). Logistic univariate analysis demonstrated that the occurrence of shunt-related intracranial hemorrhage was a significant risk factor of postoperative seizures (*P* < .001, odds ratio: 31.1, 95% CI: 6.4-151.1). When comparing patients with and without a history of previous stroke or intracranial surgery, there was a significant difference in the risk of postoperative epileptic seizures after shunt placement (**Supplemental Digital Content 1** [http://links.lww.com/NS9/A60]). Logistic regression analysis revealed that shunt-related cerebral hemorrhage was a significant risk factor of postoperative epileptic seizures in both groups. In patients with a history of stroke or intracranial surgery, the association approached statistical significance (*P* = .087; odds ratio: 23.6, 95% CI: 2.28-304). In patients without such history, shunt-related cerebral hemorrhage demonstrated a stronger association with postoperative epileptic seizures (odds ratio 31.0, 95% CI: 3.44-279.3).

**TABLE 1.** Univariate Comparison of Clinical Characteristics Between Patients With and Without Seizures After Ventricular Shunt Surgery

Clinical characteristics	Seizure (+)	Seizure (−)	*P* value
Female, sex, n (%)	4 (50)	79 (44.6)	.76
Age at surgery, median (years)	78.5	80	.28
VP shunt, n(%)	6 (75)	147 (83)	.57
Previous ICH	1 (12.8)	8 (4.5)	.38
Previous CI	2 (25%)	41 (23.1)	.90
Previous intracranial surgery	1 (12.8)	8 (4.5)	.38
Previous HT	6 (75)	85 (48)	.12
CRP (POD1), median	1.97	1.4	.65
INR	1.01	1.02	.63
APTT	33.5	31.8	.23
ICH (post shunt surgery)	5 (62.5%)	9 (5)	<.001^a^
Antithrombotic agents	2 (25)	30 (16.9)	.57
Duration of ICH from surgery, median(days)	3	7	.81

APTT, activated partial thromboplastin time; CRP, C-reactive protein; CI, cerebral infarction; HT, hypertension; ICH, intracranial hemorrhage; INR, international normalized ratio; POD, postoperative day; VP, ventriculoperitoneal.

a Statistically significant (*P* < .05).

**TABLE 2.** Univariate Comparison of the Clinical Characteristics Between Patients With ICH and Without ICH After Ventricular Shunt Surgery

Clinical characteristics	ICH after shunt surgery (+)	ICH after shunt surgery (−)	*P* value
Female, sex, n (%)	6 (42.8)	77 (45)	.87
Age at surgery, median	82.5	79	.03^a^
VP shunt, n (%)	13 (92.8)	140 (81.8)	.24
Previous ICH	0 (0)	9 (5.26)	.22
Previous CI	6 (42.8)	37 (21.6)	.09
Previous intracranial surgery	3 (21.4)	6 (3.5)	.01^a^
Previous HT	5 (35.7)	86 (50.2)	.29
CRP (POD1), median	1.94	1.4	.75
INR	1.03	1.02	.66
APTT	31.1	31.8	.74
Antithrombotic agents	7 (50)	25 (14.6)	.003^a^

APTT, activated partial thromboplastin time; CI, cerebral infarction; HT, hypertension; ICH, intracranial hemorrhage; INR, international normalized ratio; VP, ventriculoperitoneal.

a Statistically significant (*P* < .05).

**FIGURE.**
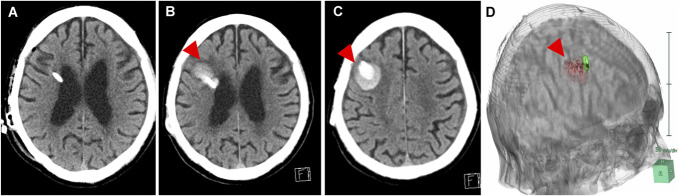
An example case of seizure due to intracerebral hemorrhage associated with ventricular shunt approximately 2 years after VA shunt surgery. **A**, CT scan immediately post-VA shunt placement. **B** and **C**, CT showed intracerebral hemorrhage around the ventricular shunt tube at approximately 2 years after VA shunt surgery. **D**, 3D reconstruction of CT images, red: hemorrhage, green: shunt tube. Arrowheads indicate the ventricular puncture site and the hemorrhagic lesion. VA, ventriculoatrial.

**TABLE 3.** Summary of Patients With VSRH

No.	Age (years)/sex	VA or VP shunt	History of ICH	History of CI	HT	previous intracranial surgery	Plt preoperative	INR/APTT (sec) preopeerative	Antithrombotic agents	CRP POD1	Onset of VSRH from surgery (days)	Seizure (±) /Onset of seizure from surgery (days)	Type of VSRH
1	80/M	VP	−	+	−	+	17.6	1.08/30.6	+	0.44	7	−	IVH
2	84/F	VP	−	+	−	−	21.4	0.99/33.3	−	0.13	7	−	Cortex
3	81/F	VP	−	+	+	−	14.4	1.06/36.9	−	0.32	7	−	Cortex
4	86/F	VP	−	−	-	−	22.1	1.03/34.7	−	3.06	3	+/3	Cortex
5	79/M	VP	−	−	+	−	9.1	1.24/48.8	−	2.82	20	+/293	Cortex
6	89/M	VP	−	+	−	−	12.8	1.07/33.3	+	1.01	14	−	Cortex
7	87/F	VP	−	+	−	−	10.3	1.04/27.3	+	1.00	1	−	SAH
8	81/M	VP	−	−	−	+	28.1	1.11/35.5	+	3.81	4	−	IVH
9	79/M	VP	−	−	+		22	1.24/48.8	+	2.61	2	+/1	Cortex
10	86/F	VP	−	+	+	−	23.9	0.94/30.8	+	2.36	1	+/1	Cortex
11	79/M	VP	−	+	+	−	18.3	1.04/28.2	+	1.53	7	−	Cortex
12	85/M	VP	−	−	−	−	18.2	0.97/29.7	−	2.74	7	−	Cortex
13	69/M	VA	−	−	−	+	15.4	0.98/31.4	−	0.55	664	+/664	Cortex

APTT, activated partial thromboplastin time; CI, cerebral infarction; HT, hypertension; ICH, intracranial hemorrhage; INR, international normalized ratio; Plt, platelet count; POD, postoperative day; SAH, subarachnoid hemorrhage; VP, ventriculoperitoneal; VA, ventriculoatrial; VSRH, ventricular shunt–related hemorrhage.

## DISCUSSION

In this study, we investigated the risk factors of seizures and shunt-related intracranial hemorrhage in patients with iNPH who underwent ventricular shunt surgery. Our results demonstrated that postoperative intracranial hemorrhage is a significant risk factor for the development of seizures after shunt surgery. In addition, older age, a history of intracranial surgery, and use of antithrombotic agents were all associated with a higher incidence of shunt-related intracranial hemorrhage. These findings align with previous studies identifying intracranial hemorrhage as a common complication of ventricular shunt surgery.

Several studies have previously identified risk factors associated with delayed hemorrhage, including advanced age, anticoagulation therapy, and history of craniotomy. In a study by Qian et al,^3^ delayed hemorrhage occurred in 7.8% of patients who underwent ventricular shunt surgery, with the use of low-molecular-weight heparin being significantly associated with a higher risk of hemorrhage. Misaki et al^4^ also reported similar findings, emphasizing that fragile brain tissue, often resulting from previous infections or trauma, may contribute to delayed hemorrhage. Li et al^7^ previously highlighted the role of inflammation in the development of delayed intracranial hemorrhage after VP shunt surgery. Our study further supports these findings, demonstrating that postoperative anticoagulation, along with factors such as advanced age, increases the likelihood of hemorrhagic complications.

In our study, CRP levels were used as an inflammatory marker. The use of CRP as a marker may have contributed to differences in findings compared with Li et al's use of the neutrophil-to-lymphocyte ratio,^7^ as CRP reflects a more general systemic inflammatory response, whereas neutrophil-to-lymphocyte ratio may be more sensitive to localized or acute inflammatory processes involved in the pathogenesis of delayed intracranial hemorrhage. This difference in inflammatory markers may partly explain the variation in our results regarding the role of inflammation in delayed hemorrhage.

Previous studies have reported varying onset times for delayed hemorrhage. For example, Qian et al^3^ observed that delayed hemorrhage could occur as early as 2 days postoperatively, with an average onset of around 4.9 days. Misaki et al^4^ reported delayed hemorrhages occurring up to 13 days after shunt surgery, demonstrating that the risk of hemorrhage persists beyond the immediate postoperative period. Our study has documented hemorrhages occurring up to 2 years after shunt placement in extreme cases, emphasizing the need for long-term surveillance. Although the exact underlying mechanism is not yet fully understood, it has plausibly been hypothesized that the fragility of the tissue at the shunt tube puncture site plays a role in precipitating cerebral hemorrhage.

The delayed hemorrhage onset highlights the importance of extended follow-up in patients undergoing shunt surgery. Patients with known risk factors, such as advanced age or anticoagulation therapy, should be monitored for longer periods to detect potential complications. In addition, comprehensive informed consent should include a discussion about the risks of delayed hemorrhage and seizures, ensuring that patients and their families are fully aware of the potential long-term risks.

Previous reports have shown an overall incidence of seizures in 9.4% of patients after ventricular shunt surgery, with a higher rate observed in younger patients and those who underwent multiple shunt revisions.^8^ In their study on seizures after intracerebral hemorrhage, Derex et al^9^ identified cortical damage from hemorrhage as a major contributor to seizure development. Similarly, our study found that patients with a history of shunt-related hemorrhage had a significantly increased likelihood of experiencing postoperative seizures. Seizure development after shunt surgery may be influenced by changes in intracranial pressure, as indicated by previous research.^10,11^ In their case study, adjusting intracranial pressure reduced seizure frequency, supporting the hypothesis that altered pressure dynamics, such as those seen with shunt dysfunction or hemorrhage, may contribute to seizure activity. In our cohort, intracranial hemorrhage likely exacerbated cortical irritation, leading to increased seizure susceptibility. Furthermore, the mechanical presence of the shunt itself, particularly in cases of migration or revision, has been implicated in seizure onset due to brain parenchyma irritation.

The association between intracranial hemorrhage and seizure development is particularly noteworthy. Previous research has shown that seizures occur in approximately 6%-15% of patients after intracerebral hemorrhage, with higher rates (up to 30%) when continuous electroencephalogram monitoring detects subclinical or nonconvulsive seizures.^9,11^ Hemorrhagic complications, particularly those involving the brain cortex, can lead to an increased risk of seizures by disrupting normal neuronal activity. Our study further substantiates this by demonstrating a significant correlation between postoperative intracranial hemorrhage and seizure occurrence. Specifically, seizures seemed to occur more frequently in patients with cortical involvement (3, which indicated that cortical hemorrhage might be more prone to seizure development compared with intraventricular hemorrhage, .These results emphasize the importance of careful postoperative monitoring, particularly in patients with identified risk factors such as advanced age, previous intracranial surgery, and antithrombotic therapy.

LP shunt surgery has also been validated as a viable and effective procedure for iNPH.^12^ However, the adoption of LP shunting as a treatment modality varies widely among institutions. In the context of this study, the choice to perform LP shunting was predominantly determined by the surgeon's individual preferences and clinical judgment. Although LP shunt surgery is associated with a risk of subdural hematoma due to overdrainage, this procedure eliminates the risk of cerebral parenchymal hemorrhage as it does not involve intracranial puncture. For patients lacking degenerative changes in the spine, LP shunt surgery may be a more favorable option. Moreover, when counseling patients regarding surgical options, it is crucial to explain the risks of ventricular shunting, such as seizure and cerebral hemorrhage, and to propose LP shunting as an alternative option for consideration.

### Limitations

This study has several limitations. First, the retrospective design may have introduced selection bias, as only patients with complete medical records were included in the analysis. Second, while the sample size was adequate to identify significant associations, it may not have been sufficient to detect less common risk factors of seizures and hemorrhage. Logistic regression analysis demonstrated that shunt-related intracranial hemorrhage was a significant risk factor of postoperative seizures, with an odds ratio of 31.1 (95% CI: 6.4-151.1). Although the wide confidence interval limits precise risk quantification, the data suggest that the odds are at least 6.4 times higher (based on the lower bound of the 95% CI), indicating a robust association. Although shunt-related hemorrhage seems to be a clinically relevant risk factor, more precise quantitative assessment will require larger case series for evaluation. Third, although cerebral microbleeds, history of trauma, alcohol use, infection, and shunt malfunction may serve as potential risk factors of intracerebral hemorrhage or seizure, we were unable to systematically evaluate these variables in this study, which represents an important limitation of our analysis. Finally, although the follow-up period extended to 2 years, it may not capture all late-onset complications. Future prospective studies with larger cohorts and extended follow-up periods are needed to further elucidate the mechanisms underlying shunt-related complications and to establish more precise risk stratification models.

## CONCLUSION

In summary, this study demonstrates that shunt-related intracranial hemorrhage is a significant risk factor of postoperative seizures in patients with iNPH undergoing ventricular shunt surgery. Careful postoperative monitoring and extended follow-up are crucial, particularly for patients with identified risk factors, such as advanced age, previous intracranial surgery, and use of antithrombotic therapy. In addition, comprehensive discussions about potential complications should be a vital component of the informed consent process, ensuring that patients and their families are fully aware of the long-term risks associated with the procedure.

## SUPPLEMENTAL DIGITAL CONTENT

**Supplemental Digital Content 1**. **Table 1**. Reanalysis of data stratified by patients with and without a history of intracranial surgery: univariate comparison of clinical characteristics (seizures vs. no seizures after ventricular shunt surgery, excluding patients with previous stroke or intracranial surgery). **Supplemental Digital Content 1**. **Table 2**. Reanalysis of data stratified by patients with and without a history of intracranial surgery: univariate comparison of clinical characteristics (seizures vs. no seizures after ventricular shunt surgery in patients with previous stroke or intracranial surgery).

## References

[1] Kobayashi E Kanno S Kawakami N Risk factors for unfavourable outcomes after shunt surgery in patients with idiopathic normal-pressure hydrocephalus. Sci Rep. 2022;12(1):13921. 35978079 10.1038/s41598-022-18209-5 PMC9385629

[2] Chen JC Duan SX Xue ZB Risk factors for delayed intracranial hemorrhage secondary to ventriculoperitoneal shunt: a retrospective study. World J Clin Cases. 2022;10(21):7302-7313. 36158027 10.12998/wjcc.v10.i21.7302 PMC9353909

[3] Qian Z Gao L Wang K Pandey S. Delayed catheter-related intracranial hemorrhage after a ventriculoperitoneal or ventriculoatrial shunt in hydrocephalus. World Neurosurg. 2017;107:846-851. 28847553 10.1016/j.wneu.2017.08.098

[4] Misaki K Uchiyama N Hayashi Y Hamada JI. Intracerebral hemorrhage secondary to ventriculoperitoneal shunt insertion-four case reports. Neurol Med Chir. 2010;50(1):76-79. 10.2176/nmc.50.76 20098034

[5] Nakajima M Yamada S Miyajima M Guidelines for management of idiopathic normal pressure hydrocephalus (third edition): endorsed by the Japanese society of normal pressure hydrocephalus. Neurol Med Chir (Tokyo). 2021;61(2):63-97. 33455998 10.2176/nmc.st.2020-0292 PMC7905302

[6] Mori E Ishikawa M Kato T Guidelines for management of idiopathic normal pressure hydrocephalus: second edition. Neurol Med Chir (Tokyo). 2012;52(11):775-809. 23183074 10.2176/nmc.52.775

[7] Li S Wang H Li F Chen M Chen P. A new inflammatory parameter can predict delayed intracranial hemorrhage following ventriculoperitoneal shunt. Sci Rep. 2021;11(1):13763-10. 34215829 10.1038/s41598-021-93315-4 PMC8253783

[8] Dan NG Wade MJ. The incidence of epilepsy after ventricular shunting procedures. J Neurosurg. 1986;65(1):19-21. 3712024 10.3171/jns.1986.65.1.0019

[9] Derex L Rheims S Peter-Derex L. Seizures and epilepsy after intracerebral hemorrhage: an update. J Neurol. 2021;268(7):2605-2615. 33569652 10.1007/s00415-021-10439-3

[10] Uchida D Fujimoto A Yamazoe T Yamamoto T Enoki H. Seizure frequency can be reduced by changing intracranial pressure: a case report in drug-resistant epilepsy. Epilepsy Behav Case Rep. 2018;10:14-17. 30062085 10.1016/j.ebcr.2017.12.005 PMC6063982

[11] Faught E Peters D Bartolucci A Moore L Miller PC. Seizures after primary intracerebral hemorrhage. Neurology. 1989;39(8):1089-1093. 2761703 10.1212/wnl.39.8.1089

[12] Kazui H Miyajima M Mori E Ishikawa M Shimada Y Ishii K. Lumboperitoneal shunt surgery for idiopathic normal pressure hydrocephalus (SINPHONI-2): an open-label randomised trial. Lancet Neurol. 2015;14(6):585–594. 25934242 10.1016/S1474-4422(15)00046-0

